# The New Regulations as to Arrears of Contributions

**Published:** 1914-02-07

**Authors:** 


### NATIONAL INSURANCE ACT DEVELOPMENTS.

### The New Regulations as to Arrears of Contributions.

Of all the alterations affecting the administration of Health Insurance brought into being by the National Insurance Act of 1913, the provisions regarding the arrears of contributions of employed contributors—the manner in which these are to be treated and the penalties for their non-payment—are among the most drastic of the alterations. The principal section of the Amendment Act dealing with the question came into operation on January 12 last, and the alterations entailed are of such importance that the Insurance Commissioners furnished to each approved society a supply of leaflets with the instruction that they were to be delivered to the members, the object being to make the altered position quite clear to all insured persons. Unfortunately this leaflet, which is very modest in dimensions, is not as explicit as it might be on many points; and it will be well, therefore, if we deal more fully with the subject on the lines of the circulars that have been issued by way of instruction to the officials of approved societies.

### Section 7.

The section of the Act referred to is that numbered seven, the effect of which is to allow employed contributors to pay up arrears of contributions due to periods of unemployment at reduced rates. Instead of having to pay the full weekly contribution—the employer's share as well as his own—as was required under the principal Act, except in such cases as the society agreed to excuse the employer's share, it is now only necessary for an employed contributor to pay the arrears of his own contributions, if he elects to pay them at all. Although this section of the Act only came into force on January 12, the Insurance Commissioners have made its operation retrospective in so far, at least, that any arrears that may be paid subsequently to that date are to be at the reduced rate, even though the weeks for which the contributions fell due were before the date mentioned.

This decision of the Commissioners to make the operation of the new arrangements retrospective is a concession that was not generally looked for, and it will, doubtless, give rise to a certain amount of dissatisfaction to those who, having fallen into arrear, have already paid up their arrears at the full rate. In justification of their action there are several important reasons that may be given. For instance, it would have raised considerable difficulties in practice if certain payments that had fallen into arrear had to be made good at one rate, while others falling due after January 12 were to be made good at the reduced rate. It would have taken much trouble to have satisfactorily explained such a proceeding to the average insured person, besides the difficulties that would have been involved in accountancy between the approved societies and the members on the one hand, and the Insurance Commissioners on the other. Thus, after all, those employed contributors who have already paid up arrears at the full rate may perhaps console themselves with the reflection that the new arrangements are conceived with a view to securing simplicity of administration, and are thus indirectly of benefit to them individually as tending to efficiency, and perhaps also as an inducement to others to pay up arrears that would otherwise not have been paid.

### How Arrears are to be Paid.

In addressing employed contributors in the leaflet already mentioned the Insurance Commissioners say, "If you wish to pay off your arrears (if any) in order to secure yourself against reduction of benefit, you should apply to your society for an arrears card." This, then, is the method of procedure. A special arrears card must be obtained, and stamps to the exact value of the contribution required should be affixed for each week that the member is in arrear. It is emphasised that it is important that a stamp of the exact denomination for the weekly contribution should be used, and as many stamps affixed as there are weeks' arrears to be paid, as this will very much simplify the working of the system, as contrasted with the use of stamps of higher individual value aggregating the same total sum. This is one of the details that employed contributors should carefully note when desirous of paying arrears.

In the normal case the amount of the stamp to be used by a male contributor on an arrears card will be 4d., and that of a female 3d., but there are some exceptions. If, for instance, the employed contributor is over age twenty-one, and does not receive board and lodging as part of his remuneration, and the rate of remuneration is less than 2s. 6d. a day, he is entitled to the benefit accorded under the principal Act to low-wage earners. That is to say, the weekly contribution deducted from his wage will be less than 4d. in the case of men, and less than 3d. in the case of women. The full scale is printed on all insurance cards and books, and it is unnecessary to reproduce it here. Suffice it to say that if a low-wage declaration has been signed by the member, or if by other means he is able to satisfy his society that the normal deduction from his wage should be at the reduced rate, then on paying up arrears due to a period of unemployment the contributor will only be required to pay at the scale applicable to the rate of his remuneration. Again, if an employed contributor is in the service of an employer who guarantees to pay full wages during sickness for the first six weeks of illness, thus entitling the insured person as well as himself to a reduction in the rate of contribution, it will be the employee's contribution, as thus reduced, that will become payable if arrears are to be paid.

In this case there does not seem to be the same justification for the reduction in the rate of contribution when paid as arrears as there is in the case of low-wage earners, for it is extremely unlikely that an employed contributor, who has been in the service of an employer who guarantees full wages during sickness, will be so fortunate as to secure another berth or situation on similar terms when he falls out of employment, and it has to be remembered that it is only while he is out of employment that arrears can become due.

### Payment of Contributions to Qualify for Benefit.

The quotation from the leaflet as given above has been very carefully worded, in that it only refers to arrears to provide against *reductions* of benefit. There is a fundamental distinction between the arrears paid for this purpose, on account of a period of unemployment, and the payment of contributions to *qualify* for benefit. The difference is somewhat intricate, but it is of importance that it should be understood. For instance, it is laid down in the Act of 1911 that arrears of contribution are not to count for the purpose of calculating the rate of benefit for any period when the member is in receipt of sickness or disablement benefit, neither are arrears to count for this purpose during the first year of the operation of the Act, that is to say, any arrears due to unemployment which fell due before July 15, 1913, do not count in the calculation of the rate of benefit. This being so, it may naturally be asked what inducement there is to pay up contributions, when their non-payment is to make no difference to the rate of benefit. The answer is that in order to qualify for sickness and disablement benefits under the Act it is necessary for 26 and 104 weekly contributions respectively to have actually been paid. It will, therefore, be seen that in many cases it is actually to the advantage of the employed contributor to pay the contribution, although he is entitled to have the payment excused.

Seeing, then, that contributions which are paid for periods of sickness, or for the first twelve months that the Act was in operation, are not arrears within the meaning of the Act, it will still be necessary for an employed contributor desirous of paying up these back contributions in order to qualify for benefit, to do so at the full rate, that is to say 7d. a week for men, and 6d. for women.

This point is not mentioned in the leaflet circulated to insured persons, but it has now been clearly laid down by the Commissioners to the approved societies that the full rate is to be paid in such circumstances. When it is desired to qualify for benefit in this way, an arrears card will not be used, but stamps must be affixed to an ordinary insurance card. If the member has such a card in his possession for the current period the stamps may be affixed thereto, or if the card has already been surrendered to his society, the member may obtain another for the purpose on making application.

It should also be clearly understood that the new regulations only refer to contributions falling into arrear through unemployment. They do not extend to the payment of contributions that may have been neglected by an employer while the contributor was at work. Such neglect to pay the contributions is a contravention of the provisions of the Acts, and if it is subsequently desired to make good the contributions that have been omitted, they will have to be paid at the full rate, as would have been the case had the stamps been affixed at the time the payment became due.

